# The Italian cross-cultural adaptation of the Social Vulnerability Index

**DOI:** 10.3389/fpubh.2025.1576223

**Published:** 2025-08-04

**Authors:** Gloria Mangini, Cristina Festari, Silvia Ottaviani, Luca Tagliafico, Gianluca Di Cara, Alessio Nencioni, Fiammetta Monacelli, Delphi Consensus Panel

**Affiliations:** ^1^IRCCS San Martino Polyclinic Hospital, Genoa, Italy; ^2^Department of Internal Medicine and Medical Specialties (DIMI), Section of Geriatrics, University of Genoa, Genoa, Italy; ^3^Laboratory of Alzheimer’s Neuroimaging and Epidemiology, IRCCS Istituto Centro San Giovanni di Dio Fatebenefratelli, Brescia, Italy; ^4^Independent Translator Specialized in Clinical Research, Genoa, Italy

**Keywords:** social vulnerability, frailty, Delphi consensus, Social Vulnerability Index, Italian adaptation, cross-cultural adaptation

## Abstract

**Background:**

Social vulnerability is a key health domain that is associated with frailty and disability in older adults, informing clinical trajectories and outcomes both on an individual and at a population level. The underlying concept is that frailty develops with the accumulation of physical, psychological, and social deficits, and the identification of losses in the social domain may allow for designing tailored interventions in a timely fashion. The aim of the present study was to adapt the Social Vulnerability Index (SVI) to the Italian language and culture for these purposes.

**Methods:**

The Italian version of the SVI (SVI-I) has been developed through a comprehensive cross-cultural adaptation of the original Canadian SVI. This process involved four steps: initial translation, synthesis of translations, back translation, and a Delphi procedure.

**Results:**

The result of the study is the face-valid 38-item SVI-I. Based on the Delphi procedure, the SVI-I can be administered to Italian-speaking, over-65, community-dwelling individuals not affected by cognitive decline.

**Conclusion:**

This study develops the first index to measure social vulnerability in the Italian-speaking population, aiming at a multidimensional approach to address social and healthcare needs. If proven effective in subsequent validation studies, it may enhance geriatric assessments, improve early social vulnerability detection, and support tailored care plans.

## 1 Introduction

Social ageing is defined by changes in a person’s social role and within their personal networks, including family and friends, and it is also shaped by societal attitudes toward ageing within society. As a result, different societal structures and functions within different countries and settings may lead to heterogeneity in the individual’s social needs or perspectives. Social relations encompass a complex and dynamic set of characteristics that have been shown to distinctly affect health and quality of life across the lifespan and especially in older adulthood (1–3).

According to the Social Production Functions theory (4) social well-being is founded upon three distinctive social needs: affection, behavioral confirmation and status. Affection is the satisfaction of the need of love and the feeling arising from it; behavioral confirmation is the sense of having acted in a manner that is perceived as appropriate by oneself and their relevant others, and of belonging to a group with shared values; status is the satisfaction with own social role due to the possession or control of socially valued resources (e.g., privileges, money, talent, power, and knowledge). Failing to meet these social needs promotes social vulnerability (SV) (5). Building on this framework, Bunt et al. categorized the key factors underlying SV into *basic social needs* such as social cohesion, social support and sense of loneliness, *social resources* such as marital or familial status or social networks and *social behaviors/activities*, including social participation, occupation, and religiosity (6). In this context, SV may be defined as the unfulfillment of at least one of the mentioned core social factors.

These theoretical frameworks provide a foundation for understanding SV as a multidimensional construct, linking the concept of unmet needs with specific, observable deficits in social connections and support. This perspective informs the definition of SV as a deficiency or scarcity in the quality or quantity of social connections, or in the degree of support, or interactions available to individuals. There is an increasing prevalence of SV, especially in the old-age population, which may be associated with adverse clinical outcomes, including functional decline, prolonged hospitalization, disability, and mortality (7–10).

With ageing, the concept of SV may partially overlap with the concept of frailty a distinct construct defined as a state of increased vulnerability to environmental stressors due to reduced physiological reserve (11). The frailty model has progressively evolved, shifting from a focus on physical dimensions to a broader, multidimensional perspective, where the progressive accumulation of physical, mental, functional and social deficits contributes to increased vulnerability to adverse outcomes (12). Similarly, SV reflects deficits in social connections, support, and resources, contributing to increased susceptibility to adverse outcomes. Given their intrinsic similarities in determinants and conceptualization, recent evidence highlighted the bidirectional relationship between frailty and SV (2, 13). A systematic review by Hanlon et al. found that SV can accelerate frailty progression, while frailty exacerbates social isolation and limits participation; furthermore, the combination of frailty and SV is associated with increased mortality, decline in physical function and cognitive performance (14).

In recent years, the concept of frailty has been progressively joined and complemented by that of intrinsic capacity (15), which focuses on an individual’s strengths and retained potential to maintain functional ability. Translating this perspective to the field of SV, there is a bidirectional interplay between SV and resilience that is acted between the individual and their environment over the life course: the dynamic interactions over social networks, access to care, and personal dynamics may shape an individual’s resilience. This empowering approach aligns with the recovery model (16), which conceptualizes recovery as a multidimensional, non-linear process across three interdependent domains: *symptomatic* (e.g., loneliness), *social* (e.g., participation and connectedness), and *existential* or *meaning-related* (e.g., autonomy, purpose, and emotional fulfillment). This model prioritizes that recovery is unique, integrating vulnerability and resilience in all aspects of life, and places importance on personal agency rather than just support.

Similarly, both frailty and SV are dynamic, modifiable constructs, that might be reversible. As societies and healthcare systems seek to respond and adapt to increasing SV and frailty at a population level, there is a need to address them at the individual level as well. This response will require a careful balance of the multidimensional and complex nature of both frailty and SV with the need for practical tools and interventions that can be implemented in healthcare settings (17, 18).

With this unifying purpose, the concept of SV has been integrated into an ecological model based on the deficit accumulation construct (12), interpreting SV as an accumulation of disadvantageous social circumstances that have negative health outcomes. A Social Vulnerability Index (SVI) is a multifactorial index that consists of a list of items including living situation, socially orientated activities of daily living, leisure activities, socio-economic status, social connections, support or interaction and housing situation. Each factor is not more important than the others, but their accumulation leads to an increase in the overall SV. The deficit accumulation model is flexible and adaptable to different settings.

Developed initially in non-medical settings (e.g., emergency management, disaster planning, or environmental hazards) (19, 20), only in the last decade SVI became popular in the medical literature. A recent scoping review by Mah et al. analyzed 121 original SVIs across various fields, purposes, and applications, identifying seven key domains: at-risk populations, education, micro-level socioeconomic markers (individual, family, or household), household composition, employment, housing, and population health statistics (21). These domains align with a socio-ecological framework (22), which considers individuals as part of nested layers of social influence. At the *micro* level, factors include individual behaviors and close ties within the family or with caregivers. The *meso* level captures interactions between these systems, such as how family or friends engage with healthcare providers. At the *exo* level, community supports like home care or rehabilitation programs play a role, while the *macro* level encompasses societal attitudes and policies, such as universal healthcare.

SVIs have been frequently used to predict outcomes, especially in healthcare and medical research. Commonly predicted outcomes include COVID-19 infection or mortality [32 studies, with significant associations in 85.1% of cases, according to the review by Mah et al. (21)], access to healthcare services or resources, and surgical outcomes. In a secondary analysis from the Canadian Study of Health and Aging, SVI was found to be able to predict mortality in older fit adults, emphasizing that changes in the health status of individuals would be secondary not only to medical conditions but also to social and environmental situations (23). Moreover, SVIs have recently been applied to the hospital setting, demonstrating a correlation, independent of frailty, with prolonged hospital stays and the need for long-term care.

It is noteworthy that the presence of highly heterogeneous social differences among older populations may hamper the mechanical application of scores developed in different contexts or may blunt the efficacy of interventions. This emphasizes the importance of the cross-cultural adaptation process, which is essential when a questionnaire is to be used in a culture, language and country other than those for and in which it was developed, to produce equivalency between source and target based on content. Indeed, this process involves not only the translation of the items, but also their cultural adaptation aiming at semantic, idiomatic, experiential and conceptual equivalence to ensure the conceptual validity of the instrument in different cultural contexts (24). This adaptation process, as outlined by Guillemin et al. (25), ranges from cases requiring minimal changes, such as use in the same language and culture, to more complex scenarios, such as applying an instrument developed in one country to a vastly different cultural and linguistic context.

A clear example of cross-cultural adaptation is the development of the Dutch Social Vulnerability Index (SVI-D) by Bunt et al. (23). This process, which will be discussed in more detail in the following sections, involved translation, synthesis, back translation, a Delphi procedure, and testing for face validity. The resulting 32-item index excluded culturally irrelevant items, while retaining the broad applicability of the original SVI. Details of the collaborators of the Delphi Consensus Panel are available in Supplementary materials S1.

Italy is one of the oldest countries in the world, with over 23% of its population aged 65 years and above (26). The ageing population is associated with profound shifts in social structures: about one-third of older adults live alone, and traditional family-based care systems are being reshaped by declining fertility, increased mobility, and changing household compositions (27). Although ageing in Italy has been characterized by intergenerational ties, with older adults having active roles in family and community as keepers of collective memory and cultural heritage (28, 29), substantial transformative issues are permeating the society. Namely, geographic inequalities are growing, with central and northern Italy having well-developed societal networks with higher care access, and southern regions suffering from more fragmented societal networks and services. Similarly, community networks show disparities through Italy; in this context, grassroots initiatives such as the Community of Sant’Egidio, founded in Rome in 1968, play a key role in sustaining SV through the provision of social support and services devoted to older frail adults through proactive monitoring, community engagement, and rapid-response systems, particularly in areas with high levels of SV. Its project “Viva gli Anziani!” addresses social isolation and supports over 28,000 older adults across Italy through active monitoring, community engagement, and rapid response to critical events like heat waves.

Based on this background, and on the absence of reliable instruments to detect SV in the Italian older population, the aim of the study is the Italian cross-cultural adaptation of the SVI for use in the Italian context.

## 2 Methods

**Figure 1:**
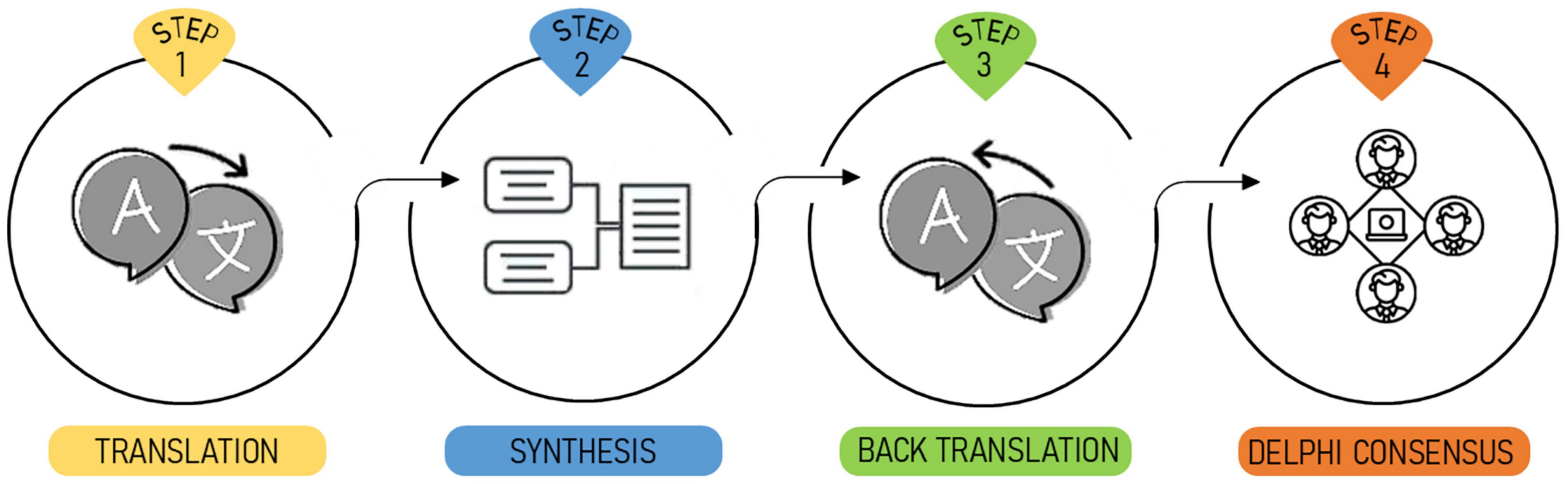
Graphical representation of the steps of the theoretical framework used for the cross-cultural adaptation of the SVI-I.

To adapt the SVI to the Italian language and culture, we used guidelines for cross-cultural adaptation of measurement instruments (30), briefly summarized in Figure 1. This study was approved by the IRB (CERA N 2024-54 12/06/2024) of the University of Genoa, Italy.

### Step 1. Translation

In the first step, two professional native Italian translators with fluent English independently translated the 40 items of the SVI from the Canadian Study of Health and Aging (CSHA) and the 23 items from the National Population Health Survey (NPHS). All items were considered potentially relevant at this stage. The first translator is a long-term experienced linguist specialized in technical-scientific translation, resident in Genoa; the other translator is an academic with a PhD in Philosophy, currently residing in Australia. The entire translation process was conducted in accordance with ISO 17100:2015, the international standard for professional translation services (31).

### Step 2. Synthesis of the translations

These two Italian versions of the Canadian SVI were reviewed and summarized under the supervision of an experienced geriatric researcher. Considering 40 items from the CSHA and 23 items from the NPHS both in T1 and T2 versions, the working group selected 63 of 126 items identified as the simplest and clearest. Subsequently, the working group decided to keep the original formulation of 21 items, to introduce *de novo* 9 items, to merge 6 items into 3 items and to modify 2 items. Finally, 34 items were removed because they were deemed repetitive or not suitable. This preliminary version of the Italian SVI consisted of 35 items. Details of the synthesis process are available in Supplementary materials S2.

### Step 3. Back translation

The Italian 35 item version was back-translated into English by two native English speakers fluent in Italian. This back-translation was compared to the original SVI to identify any changes in meaning that may have occurred during the translation process. Relevant discrepancies were discussed and resolved by consensus.

The back-translations were performed by a bilingual (English/Italian) medical doctor specialized in geriatrics, and by a US-born medical researcher who has resided in Genoa for several years. Although back translation is not a formal requirement under ISO 17100, it was implemented as an additional quality assurance measure.

### Step 4. Delphi consensus

The final step consisted of a Delphi procedure, a group facilitation technique used to achieve a shared opinion or decision by surveying a panel of experts (i.e., the panelists) through a series of structured questionnaires, commonly referred to as rounds. For this project, a modified Delphi procedure was used (32, 33). Prior to initiating the first round, a virtual kick-off meeting was held with all panelists. During this session, the research team presented the objectives of the study, the structure of the Delphi process, and the criteria guiding the evaluation of the items. Particular attention was given to explaining the anonymized nature of data handling, the rationale behind the adaptation process, and the procedures for reaching consensus. This initial meeting ensured a shared understanding of the methodological framework, while preserving the independence of expert judgment throughout the subsequent phases. After the meeting, each panelist received an invitation via email with access to the web-based platform (34),^1^ where they were asked to complete a structured questionnaire evaluating the preliminary version of the Italian SVI. The panelists were given two weeks to provide their opinion on the suitability and relevance of each item in Italian culture. Justification of each response was mandatory in order to support transparency and allow meaningful aggregation of feedback. Panelists were allowed to abstain from voting. We defined an *a priori* threshold of 70% of non-abstaining voting panelists for agreement. When a question needed re-discussion, an absolute majority (50% + 1) was deemed sufficient for agreement (35). After each round, anonymized summaries of the distribution of responses and the accompanying justifications were compiled by the research team and shared with the panel. A detailed explanation of the decisions made—such as item retention, rewording, or exclusion—was also provided, ensuring full transparency in the iterative refinement process. For this project, three Delphi rounds were performed to reach an agreement for the final version of the Italian SVI. Consistent with the principles of the modified Delphi method, no interaction occurred among panelists during or between the rounds. All exchanges took place exclusively between individual panelists and the research team, thus safeguarding the independence of expert opinions and minimizing the risk of influence or group pressure.

A panel of 28 experts in the field of frailty and ageing, working at different hospitals and healthcare facilities across Italy, was established to achieve a consensus on the final items of the questionnaire. Experts were recruited by invitation through internal communication channels of two national geriatric societies, without any preliminary selection. These experts were deemed to possess valuable knowledge of the concept of SV and the suitability of the items for measuring this concept in the Italian context. In order to provide the perspective of older adults, a voluntary association (“Viva gli Anziani!”) that focuses on SV in the geriatric community was invited to participate as an additional panel member.

## 3 Results

### 3.1 Features of the Delphi panel members

The panel comprised 17 geriatricians, 11 psychologists and 1 voluntary association member. A total of 89% of the panel members (15 geriatricians and 10 psychologists) indicated that they were engaged in scientific research pertaining to healthy and pathological ageing. All participants had extensive professional experience, with a mean± standard deviation of 19 ± 12 years for geriatricians and 13 ± 7 years for psychologists. The majority of panelists identified as experts in frailty (86%) and/or SV (36%). The panelists reflected those who responded to the invitation and were mostly based in northern and central Italy, with only two experts from the southern regions.

### 3.2 The Delphi procedure

**Figure 2:**
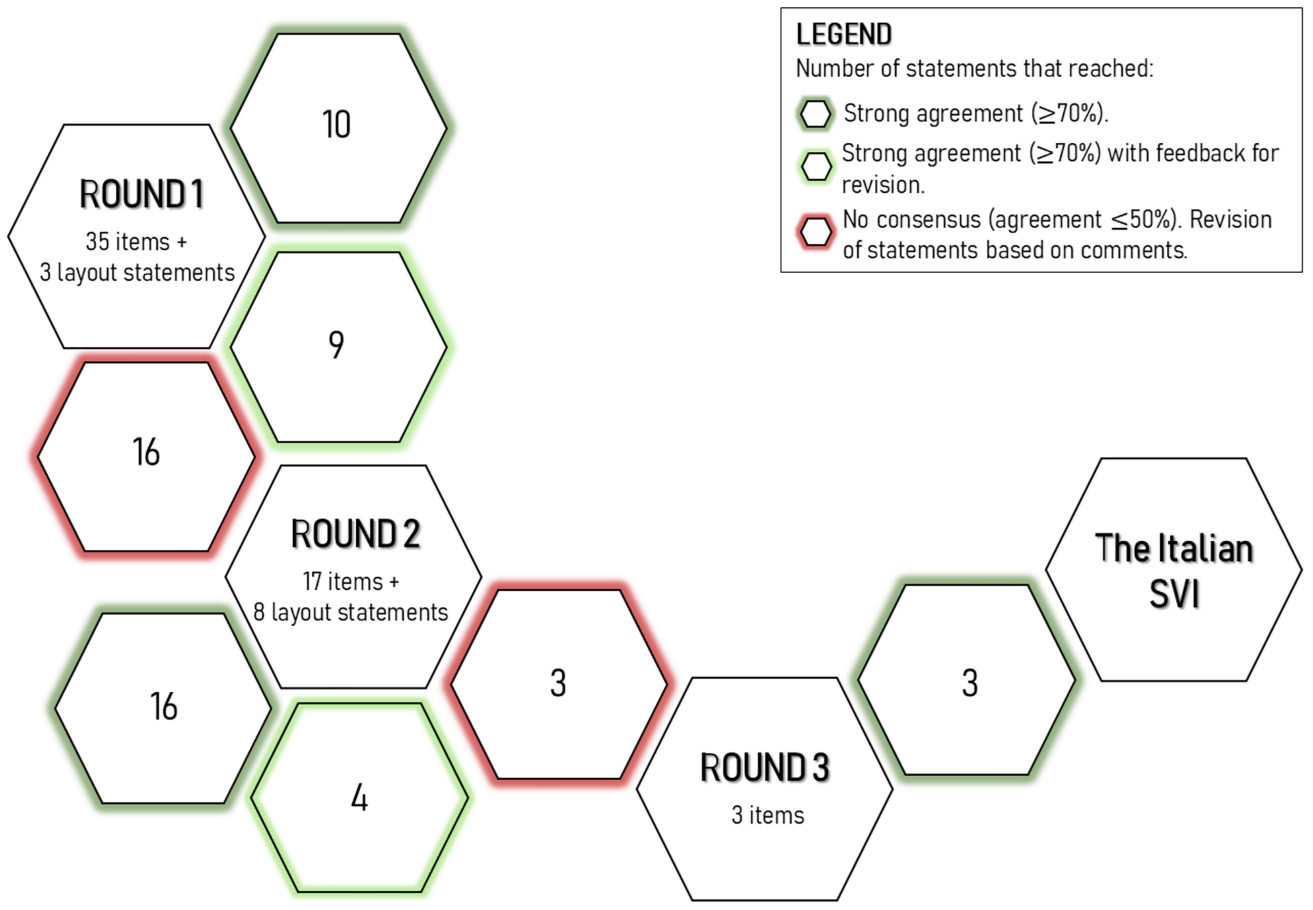
Overview of the Delphi procedure. In three rounds, 38 items and 11 statements about the layout were discussed. The figure illustrates the content of the Delphi questionnaires, indicating the number of statements discussed and the percentage of agreement.

The Delphi procedure consisted of 3 Rounds, as summarized in Figure 2.

#### 3.2.1 First Delphi round

The first Delphi round was launched on 14 March 2024, at the end of a kick-off meeting conducted online in synchronous mode. In the first questionnaire, the panel was tasked with evaluating the conceptual relevance and wording of the preliminary version, including 35 items subdivided into 7 sections, in the Italian context. The panel had five options for each item, including keeping the item as it is, reformulating it, modifying the delivery, modifying the response options, or deleting it. Each response required justification. In the second section of the questionnaire, experts were given the opportunity to change the titles and order of the sections and to propose new entries.

All 29 experts completed the questionnaire. Of the 35 proposed items, 16 did not reach the minimum percentage of consensus, while 19 items reached consensus (i.e., items 4, 6, 7, 10, 12, 13, 15, 16, 17, 18, 19, 26, 29, 30, 31, 32, 33, 34, and 35). However, the wording of 9 of these items was changed insubstantially (i.e., items 4, 7, 10, 13, 15, 16, 19, 26, and 31) based on the comments received.

The Panel proposed amendments to the title of section 1 and a merger of sections 2 and 3. Furthermore, the Panel put forth 3 additional items for consideration. These comments were discussed in Delphi Round 2.

#### 3.2.2 Second Delphi round

The second Delphi Round convened on 28 March 2024. The experts were requested to re-examine the items that had not reached a consensus, contemplating the anonymous outcomes of the initial round. They were asked to assess the suitability of each item for inclusion and the appropriateness of its wording in the pre-final index. Furthermore, qualitative feedback was required to substantiate their response.

All the 29 panelists completed the questionnaire and 14 of the 16 items reached the consensus (i.e., items 3, 5, 6, 8, 9, 11, 14, 20, 21, 22, 23, 24, 25, and 28). However, the wording of items 14, 21, 23 and 28 was modified in accordance with the group’s recommendations. The proposed amendments to the sections (i.e., title or order) were met with consensus. Indeed, 73% of the panel members endorsed the proposed title for Section 1 (i.e., “Communication Skills”), and 79% concurred with the suggestion to combine Section 2 with Section 3. All 3 items proposed in the first round were approved for inclusion in the SVI-I. In particular, item A, which pertains to the frequency of utilization of digital devices as communication tools, attained 86% consensus; item B, which concerns the frequency of participation in cultural activities, attained 79% consensus; and item C, which pertains to the accessibility of medical care services, attained 86% consensus. Furthermore, the panel achieved consensus on the sections into which the new items should be integrated.

#### 3.2.3 Third Delphi round

In the third round, the remaining items that had not reached a consensus were discussed (items 1, 2, and 29). The participants were invited to select one of two proposed wording options or to abstain. The questionnaire was distributed on 2 May 2024 and was completed by 28 experts. The final wording of the items obtained moderate agreement, with a range of 54 to 61%. This level of agreement was considered sufficient as it was a re-discussion of the items.

Additionally, qualitative feedback was gathered regarding the questionnaire layout and instructions for examiners.

The final iteration of the SVI-I consisted of 38 items. The percentage of agreement for each item is shown in Supplementary materials S3.

### 3.3 Final structure of the SVI-I

The final version of the SVI-I consists of six sections:

#### 3.3.1 Communication skills

This section includes 2 items, which reached agreement rates of 54 and 57%, respectively. These items were extensively debated across all rounds and only reached consensus in the third round through a majority vote. Some panel members argued that “reading proficiency in Italian” is a prerequisite for completing the questionnaire and, as such, should not be included in the final score. Others countered that, while the Italian translation may not fully reflect the bilingual context of the original Canadian version.

Moreover, questions on “reading and writing ability” in Italian remain relevant—particularly in cases of low literacy or for non-native Italian speakers.

The final items included are: “*How do you rate your ability to read in Italian?*,” and “*How do you rate your ability to write in Italian?*”

These items are especially important for identifying educational disadvantages or potential sensory impairments. To account for such challenges, interviewers may offer reading assistance to ensure accurate and complete responses.

#### 3.3.2 Social support

This section consists of 14 items, all of which received a high level of agreement, ranging from 72 to 93%. The item with the highest consensus was: “*Do you have someone you can rely on for transportation?*” (93%). One of the lowest was: “*Do you feel you need more support with important matters (*e.g.*, unexpected expenses, medical decisions)*?” (72%).

The most debated item was: “*Who do you live with?*.” Panel members discussed appropriate wording for response options, debating whether to include terms like “*husband*” and “*wife*” versus more inclusive terms like “*partner*” or “*spouse*.” Others emphasized the importance of recognizing emerging living arrangements such as co-housing or family-style homes, even if they are not yet common-use in Italy, as well as the presence of live-in caregivers.

#### 3.3.3 Daily activities in social contexts

Composed of 4 items, this section had agreement rates between 72 and 90%. The item with the highest agreement was: “*Are you part of any volunteer organizations or other groups?*.” The lowest agreement was for: “*Do you use the telephone?*.” A new item was introduced concerning the use of digital tools for communication, accessing information, and telemedicine. Another item—regarding the perception of being useful to the community—was adapted from the original Canadian version, with the addition of the family context, pointing out the key relevant role of older adults in continue supporting children and grandchildren.

#### 3.3.4 Recreational activities

This section includes 13 items, with agreement rates ranging from 61 to 93%. The highest-rated items were: “*How often do you visit clubs, religious centers, community centers, or other social gathering places?*,” “*Do you play cards or other board games?*” (both 93%). The lowest agreement was for: “*How often do you engage in outdoor activities (*e.g.*, gardening, fishing, going to the park)?*” (61%). Panel members discussed which types of activities should be included extensively. A consensus was reached to classify activities into three distinct groups: outdoor, at-home, and physical activities. Examples of such activities were tailored to Italian older adults’ common practices, including gardening, dancing, and solving crossword puzzles. Moreover, the participation in cultural events, championed by groups like *Viva gli anziani!* was also introduced along with the presence of a living pet that was consider to play a protective role on social isolation and loneliness.

#### 3.3.5 Psychological well-being

This section included 5 items, with agreement rates varying between 61 and 86%. The most controversial item was: “*Do you feel you can freely make decisions about important matters in your life?*,” which reached consensus only in the final round by majority vote. Critics found the item vague and potentially difficult for older adults—especially those who are socially vulnerable—to interpret. Nevertheless, this domain is underrepresented in the original version, as several items were considered more appropriate for younger individuals with greater introspective abilities.

The majority ultimately supported the inclusion of the item, emphasizing the importance of self-determination and autonomy for older adults’ well-being. This view aligns with the principles of Integrated Care for Older People (ICOPE), a framework developed by the World Health Organization to promote healthy aging. ICOPE emphasizes the empowerment of older individuals, encouraging them to make informed decisions and set their own health goals (36).

Other items in this section assess the capacity to form deep, trusting relationships, and the feeling of being cared for and loved—aspects also addressed in ICOPE’s care pathway (e.g., “*Do you have close relationships?*,” “*Do you often feel lonely?*”).

The final item—“*Do you feel satisfied with your life?*”—was criticized for overlapping with a commonly used instrument to assess depression, the Geriatric Depression Scale (37). To address this concern, the adverb “*currently*” was added to prompt respondents to focus on their present state rather than recalling past life experiences.

#### 3.3.6 Socio-economic situation

This section includes 5 items, which reached high agreement rates, ranging from 83 to 97%. The item with the highest consensus was: “*Do you own your home?*” (97%). This result reflects the strong cultural significance in Italy of homeownership as a key indicator of social stability and social reserve. Overall, the agreement rates in this section were consistently high, even for the item with the lowest consensus: “*Does your current income meet your needs?*” (83%).

The ICOPE care pathway similarly emphasizes the assessment of the domestic environment—asking questions such as “*Do you have problems with your home, for example house condition, location, safety?*”—and financial situation, with items like “*Do you often have insufficient funds to pay for your food, housing, and health care costs?*.” (36) Importantly, ICOPE focuses more on the perceived adequacy of resources relative to needs, rather than on the actual income amount, aligning with the approach taken in this section.

## 4 Discussion

This study presents the Italian adaptation of the SVI, a tool aimed at assessing social components of health status, predicting outcomes and potentially designing social interventions for community-dwelling older adults. Due to its self-administered format, the SVI-I is intended for individuals without cognitive impairment, to ensure adequate comprehension and response accuracy. The multi-component nature of the items in the SVI-I supports a comprehensive and multidimensional identification of aspects of SV, tailored to Italian social ageing and enabling the capture of elements of sociality not foreseen in the original version.

One of the challenges in advancing SV research is precisely the significant heterogeneity in its definition and measurement across studies, which complicates cross-cultural comparisons and interventions. Cultural diversity—reflected in variations in ethnicity, behaviors, family structures, and social ties—plays a crucial role in how SV is experienced and managed. To address these differences, SV assessment tools, such as SVI, require cultural adaptations that respect and reflect intercultural nuances.

To tailor the original SVI to the Italian context, we approached cross-cultural adaptation by carefully evaluating the cultural relevance of each item and incorporating suggestions from the expert panel. In the cross-cultural adaptation of the original SVI, a few items that did not apply to the Italian context (e.g., items relating to bilingualism or to activities that are not as common in Italy as they are in Canada, such as playing golf) were discarded before the Delphi procedure.

On the other hand, three items were introduced *de novo* in order to emphasize the importance of having satisfying relationships with family members and loved ones, as well as the importance of feeling useful to the family and the community, having adequate resources for material needs, receiving care and attention that responds to one’s psychological needs, and taking care of a pet. Five members proposed to explore the use of digital tools to keep in touch with other people (e.g., video calls and social media), to gather information and to get social and health services. In Italy, internet usage and digital skills among older adults remain low, with only 19.4% of those aged 65–74 possessing basic competencies and 53.4% of old-age-only households having internet access. Barriers include lack of digital literacy, regional disparities, and educational gaps, highlighting the urgency of inclusive digital initiatives (27). This digital divide exacerbates SV among the older adults, limiting their access to essential services and full participation in an increasingly digital society.

Two members noted the absence of items regarding cultural activities (e.g., libraries, theater, university of the third age, trips and travels). Literature data indicate that cultural activities, such as active theater participation, enhance the older adults’ social competencies, including empathy, communication, and social interactions (38). These activities also contribute to improved social well-being, reduced isolation, and better mood, while supporting cognitive health. However, barriers to access, particularly for people with physical or sensory disabilities, remain a challenge (39).

Eventually, three members proposed the inclusion of an item concerning the accessibility of community health care services (e.g., general practitioner, home care, specialist medical examinations). Access to healthcare services for older adults in Italy is often hindered by geographical, economic, and infrastructural barriers. Seniors, particularly those in southern and rural areas, face challenges such as long waiting lists, limited transportation options, and financial constraints, which can delay or prevent necessary medical care (40).

Other proposals concerning polypharmacy, quality of sleep, number of children, and presence of subjective memory deficit were not introduced as they are already being explored in other domains of the comprehensive geriatric assessment.

To further enhance the conceptual framework of SV within the Italian context, it was necessary to critically evaluate its broader implications. Beyond identifying specific risk factors, the adaptation of the SVI aimed to delve deeper into the underlying dynamics of SV, a multifaceted construct that requires a holistic understanding, especially as it may accelerate individuals’ progression toward frailty. For instance, the concept of SV has traditionally been linked to generic social support, though recent insights underscored that it is not simply the presence of support that matters, but rather the quality, emotional depth, and satisfaction derived from one’s relationships (41). Excessive or overly intensive support can, in fact, foster dependency and lead to *learned helplessness* (42), where individuals may lose autonomy and the motivation to manage their own needs. This suggests that SV should be reframed with a more positive, empowering perspective that emphasizes resilience and autonomy.

The items supporting this thesis are varied. For instance, Item 3 highlights the concept of living with non-family “*friends*.” The panel members endorsed retaining the term “friends” in the response options, recognizing that protective relationships against SV extend beyond family life. In the “*Social Support*” section, items 13, 15, and 16 explore the quality of social relationships, including those with neighbors, and the level of satisfaction with these connections.

The potential for receiving assistance with daily activities is assessed through items 4, 6, and 8, while the adequacy and sufficiency of such help in addressing needs are addressed in items 5, 7, 9, 10, 12, and 14. The next section examines the ability to stay connected with others via digital tools, participate in volunteer groups, and experience a sense of usefulness within one’s community (items 17, 18, 19, and 20).

Lastly, the “*Psychological Well-Being*” section is particularly significant. It delves into the sense of autonomy, the ability to make decisions on important life matters, and the experience of feeling loved and cared for within trusting relationships that allow for freedom of choice in personal affairs (items 29, 30, 31, 32, and 33).

### 4.1 Strengths and limitations

A notable strength of this study is the systematic approach taken to adapt the SVI to the Italian language and cultural context. The integration of a Delphi procedure proved to be a valuable addition, being a well-known methodology that allows experts to express their opinions freely. The expert panel consisted of experts from clinical medicine and social sciences, and a voluntary association. This provides a broad scientific and professional scope on the potential relevance of the items included in the SVI-I. Moreover, the experts’ high adherence rate to the Delphi procedure of the experts (i.e., drop-out rate: 4%) strengthened the results of this study. To maintain the panelists engaged the panelists, we contacted them directly and explicitly outlined the requisite commitment for the process. We repeated the initial meeting twice to reinforce their participation and conducted the Delphi procedures within a clearly delineated timeframe.

On the other hand, there are some limitations that advocate for caution in the adoption of the SVI-I in clinical practice. Firstly, as outlined by Beaton et al. (30), the final stage of the adaptation process is the pretest, conducted on 30–40 subjects in the target setting, with the objective of obtaining a measure of the quality of content validity. It will be necessary to test the SVI-I in Italian adults over the age of 65 in the future to assess its construct validity, reliability and psychometric properties. Secondly, the number of social components included in this present Italian version may have different relative weights on SV and its associated healthcare outcomes in older adults. While the panel included experts from different regions, representation was more concentrated in central and northern Italy, which may have influenced some perspectives. Future analyses of single items or cluster of items driving association with outcomes will provide specific information on social determinants of health.

### 4.2 Future directions

Future research efforts are needed to validate the SVI-I and support its implementation in clinical and community settings.

An ongoing pilot study is evaluating feasibility, acceptability, and interrater reliability in primary care and community settings, with participant and rater feedback guiding item refinement. A focus group with stakeholders will address implementation challenges and future directions.

Similarly, collaborative partnerships with healthcare providers, social services, and community organizations will be essential to ensure a meaningful implementation of the instrument.

To engage end users such as geriatric services, a second pilot will investigate correlations between SV and clinical dimensions such as frailty, mood, functional autonomy, nutrition, multimorbidity, and polypharmacy, to foster the developmental process for routine clinical use, adapting the instrument to real-world Italian old-age populations.

Given the high incidence of cognitive impairment in older populations, future developments may include an adapted version of the SVI-I to be completed by caregivers, focusing solely on observable aspects of SV. In addition, a version specifically designed for use in nursing homes or assisted living facilities is under consideration to assess SV in those care environments.

Ultimately, broader multicenter studies across Italy are needed to validate the SVI-I and promote its integration into routine geriatric care, aiming to enhance social service activation, volunteer engagement in high-risk areas, and home-based care for vulnerable populations.

## 5 Conclusion

The main result of this study is the development of an index allowing the quantification of SV in Italian-speaking countries, offering a multidimensional approach to encompass the complexity of social circumstances and holding potential for tailored interventions and prediction of healthcare outcomes both at an individual level and at a population level. This adaptation fills a critical gap in the Italian healthcare and social support system by providing a standardized measure that is both scientifically rigorous and culturally relevant.

If the SVI-I proves to perform well in clinical practice it could be widely adopted in different settings. The integration of the SVI-I into routine geriatric assessments could enhance early detection of SV, enabling tailored care plans that address both medical and social determinants of health. Furthermore, its multidimensional approach offers an opportunity to explore the interconnections between SV and frailty, paving the way for more comprehensive strategies in managing ageing populations.

## Data Availability

The raw data supporting the conclusions of this article will be made available by the authors, without undue reservation.

## Footnotes

^1^ https://ec.europa.eu/eusurvey/home/welcome
