# Supplementary material for: The Italian cross-cultural adaptation of the Social Vulnerability Index
Source: Front Public Health. 2025 Aug 4;13:1576223. doi: 10.3389/fpubh.2025.1576223 (PMC12358436; doi:10.3389/fpubh.2025.1576223)
Supplement: Supplementary file 1 [file Table_1.docx]

**SUPPLEMENTARY MATERIALS SVI-I**

**S1 Delphi Consensus Panel**

Giuseppe BELLELLI, School of Medicine and Surgery, University of Milano-Bicocca and Acute Geriatrics Unit, Fondazione IRCCS San Gerardo dei Tintori, Monza, Italy

Federica BELLONE, Department of Clinical and Experimental Medicine, University of Messina, Messina, Italy.

Mario BO, Geriatric Unit, Department of Medical Sciences, University of Turin, Città della Salute e della Scienza, Molinette, Turin, Italy.

Stefano BOGGI, Fondazione Opere Pie Riunite di Codogno ONLUS, Lodi, Italy.

Laura BOLOGNA, UOS Ospedale -Territorio, referente CDCD, UOC Geriatria AULSS 7-ALTO VICENTINO, Vicenza, Italy.

Cristina BONOMINI, U.O. Alzheimer, IRCCS-Istituto Centro San Giovanni di Dio Fatebenefratelli, 25125 Brescia, Italy.

Melania CAPPUCCIO, ASST Bergamo Est, distretto di Albino Pradalunga,Bergamo, Italy

Valeria CALSOLARO, "Geriatrics Unit, Department of Clinical and Experimental Medicine, University of Pisa, Via Paradisa, 2, 56124, Pisa, Italy.

Carlo CUSTODERO, Clinica Medica “A. Murri”, Department of Precision and Regenerative Medicine and Ionian Area (DiMePre-J), University of Bari “Aldo Moro”, Bari, Italy.

Marina DE RUI, Department of Medicine (DIMED), Geriatrics Unit - University Hospital of Padova, Padova, Italy

Maria DE VITA, Department of General Psychology (DPG), University of Padua; Geriatric Unit, Department of Medicine (DIMED), University of Padua.

Elena FIPALDINI, UOC Geriatria AULSS 7-ALTO VICENTINO, Vicenza - Italy,

Marika FERRACCI, Orthogeriatric and Geriatric Units, Gerontology and Geriatrics Section, Department Medicine and Surgery, Geriatric Institute, University of Perugia Medical School, S. Maria della Misericordia Hospital, C Building, 4° Floor, Room 20, S. Andrea delle Fratte, Perugia, Italy.

Vincenzo GALLUZZO, Center for Geriatric Medicine (CEMI), Fondazione Policlinico Universitario "Agostino Gemelli" IRCCS, Largo A. Gemelli 8, 00168, Rome, Italy.

Anna Giulia GUAZZARINI, Division of Gerontology and Geriatrics, Department of Medicine and Surgery, University of Perugia, Santa Maria della Misericordia Hospital, Piazzale Menghini 1, 06129 Perugia, Italy.

Alessandra MARENGONI, Department of Clinical and Experimental Science University of Brescia, Brescia, Italy

Sara MONDINI, Department of Philosophy, Sociology, Education and Applied Psychology (FISPPA), University of Padova, Italy. Via Venezia, 14 (35131) Padova; IRCCS San Camillo Hospital, Via Alberoni, 70 Lido - Venice 30126 (Italy)

Sonia MONTEMURRO, Department of Philosophy, Sociology, Education and Applied Psychology (FISPPA), University of Padova, Padua, Italy

Francesca MORGANTI, Department of Human and Social Sciences; CHL - Centre for Healthy Longevity, Università di Bergamo, Bergamo, Italy

Enrico MOSSELLO, Department of Experimental and Clinical Medicine, University of Florence, and Division of Geriatric Medicine for High Intensity Care, Careggi University Hospital, Florence, Italy

Ilenia MURASECCO, Division of Gerontology and Geriatrics, Department of Medicine and Surgery, University of Perugia, Santa Maria della Misericordia Hospital, Piazzale Menghini 1, 06132 Perugia, Italy.

Chiara MUSSI, Department of Biomedical, Metabolic and Neural Sciences, O.U of

Geriatrics, University of Modena and Reggio Emilia, Italy

Bruno PERNICE, Department of Medicine, University of Verona, Verona, Italy.

Andrea ROSSI, Treviso, Italy, Department of Medicine, Section of Geriatrics, Aulss 2 Treviso; Nursing and Midwifery, School of Medicine, University of Padua

Carmelinda RUGGIERO,Orthogeriatric and Geriatric Units, Gerontology and Geriatrics Section, Department Medicine and Surgery, University of Perugia Medical School, S. Maria della Misericordia Hospital, S. Andrea delle Fratte, Perugia, Italy.

Giulia SALERNO TRAPELLA, UOC Geriatria Ospedale Alto Vicentino Santorso ULSS7, Vicenza, Italy

Giulia SEBASTIANUTTO, Department of Philosophy, Sociology, Education and Applied Psychology (FISPPA), University of Padova, Italy. Via Venezia, 14 (35131) Padova, Italy

Alessandra ZIVELONGHI, UOC Geriatria B Azienda Ospedaliera Universitaria Integrata, Verona, Italy

Program “VIVA GLI ANZIANI!” Comunità di Sant’Egidio Italy

**Other collaborators:**

Francesco LANDI, Rome, Italy

Patrizia MECOCCI, Perugia, Italy

Giuseppe SERGI, Padua, Italy

[Mauro ZAMBONI, Verona, Italy](https://www.medicina.univr.it/fol/?ent=persona&id=1370&lang=en)

**S2 Cross-cultural adaptation and Delphi’s procedure**

We discarded for the purpose of cross-cultural adaptation the following items:

- “How often do golf or play other sports”,
- “Get to places out of walking distance”,
- “Can speak English or French”,
- “Neighborhood or community is too noisy or polluted”.

We removed the following items because considered difficult to understand in a self-reported scale for older people:

- “People would describe me as a giving person”,
- “Too much is expected of you by others”,
- “You would like to move but cannot (control/empowerment)”,
- “Feel that you are a person of worth at least equal to others”,
- “You take a positive attitude toward yourself”,
- “How often have people you counted on let you down”.

We modified:

“How often work in the garden” and “How often go out for a walk” extending the concepts into "working outdoors" and “going out for physical activity” and specifying in brackets various activities suitable for the habits of our old-age population (garden, vegetable garden, fishing and ballroom dancing, sports and physical activity, walks).

We merged:

- “Marital status” and “Lives alone”
- “Someone to count on to listen” and “Someone to turn for advice”,
- “How often go for a walk” and “How often golf or play other sports”.

We introduced “de novo”:

- “Are you satisfied with your family relationships”,
- “Has good relationships with your neighbors”,
- “Is involved in volunteer organizations or other groups”,
- “Does he/she feel useful to his/her family and/or the community”,
- “How often do you do activities at home (e.g. DIY, crosswords, knitting)”,
- “Does he/she take care of a pet”,
- “Has someone who makes you feel loved and takes care of you”,
- “Are you satisfied with your life”,
- “Is your home adequate for your needs”.

We retained the following items, but we reformulated them as suggested by panel members:

- “Someone to count on for help or support”,
- “Feel need more help or support”,
- “Someone to count on for transportation”,
- “Feel need more help with transportation”,
- “Someone to count on for help around the house”,
- “Feel need more help around the house”,
- “Feel need more people to talk with”,
- “Number of people spend time with regularly”,
- “Feel need to spend more time with friends/family”,
- “Feel need more advice about important matters”,
- "Telephone use”,
- “How often visit friend or relatives”,
- How often go to clubs, church, community centre
- How often play cards or other games
- “Feel empowered, in control of life situation”,
- “Maintaining close relationships is difficult and frustrating”,
- “Experience of warm and trusting relationships”,
- “Does income currently satisfy needs”,
- “Homeownership”,
- “Education”.

Finally, we eliminated the items repeated in the two versions and those expressed in domains already represented.

During Round 2, the elimination of section 2 “Housing situation” was approved by converging the item in Section 3 “Social support” and the rewording of Section 1 in “Communication skills” was approved.

Furthermore, 3 new items were proposed (A, B and C inserted respectively in the Sections “Daily activities in the social sphere”, “Recreational activities” and “Socio-economic situation”).

- ITEM A. “Do you use digital tools (smartphone, tablet, PC) to communicate (e.g. social media, video calls, instant messaging), get information (online news, debates, political participation) and/or receive medical support? Answer options: Yes/Some/No”
- ITEM B: “How often do you take part in cultural activities (e.g. third-age university, theater, musical activities, travel...)? Answer options: Never/Rarely (less than once a month); Sometimes (less than 4 times a month); Often (4 or more times a month)”
- ITEM C: “Do you feel you have adequate access to care (general practitioner's office, specialist visits, home care and nursing care)? Answer options: Yes (all services); Quite a bit (only some services); No (none or almost no services)”

These additional components have a relevant importance in the current social Italian dimensions, allowing for higher connections and fulfillment of social needs.

The panel members also expressed an opinion on the response options of some items, in particular among the responses to item 3 “Who do you live with?” new housing solutions for the elderly Italian population such as co-housing were introduced. However, the majority of the panel expressed themselves in favor of keeping the spouse among the options, although more generic formulations such as “with partner” were proposed. Finally, “live-in care staff” was included among the options, while informal hourly support was considered implicit in other items (e.g. item 4 “You can count on someone when you need help with daily activities”).

Three members of the panel expressed doubts about the wording of some items that would be of equal meaning (for example item 8 and item 9). This structure, however, has been maintained since in the SVI emphasis is given not only to reality data but also to perceived needs (e.g. despite having someone to count on to look after the house the person could still perceive the need to receive more help).

The response options relating to the frequency of recreational activities were also much discussed [Never/rarely (less than once a month), Sometimes (less than 4 times a month), Often (4 or more times a month)]. Two panelists believe the proposed frequencies are not meaningful.

14 items received non-substantial changes (9 in Round I and 5 in Round II). The intent is to generate less frustration in the subject in the event of a negative response. Other non-substantial changes concerned the activities listed for example in brackets.

Among the options of item 37 “What is your educational qualification?”, the inclusion of the university qualification differentiated from the High School qualification was approved to represent a growing number of people over 65 who have a degree as well as a higher education qualification, while elementary and middle schools were merged.

The final domains of SVI-I are similar to the original version, but “Living situation” has been included in “Social Support”, “Communication to engage in wider community” has been modified in “Communication Skills” and “Ryff Scale” has been translated in “Well-being Scale”. “Social support”, "Social oriented Activities of Daily Living”, "Leisure Activities”, “Socioeconomic status” have been retained.

**S3** This table summarizes the final version of the SVI-I. For each item, the number of voters, the percentage of agreement and the round in which agreement was reached are given.

| **Item** | **Voting panelists** | **Round** | **%**  **agreement** | **Abstain** |
| --- | --- | --- | --- | --- |
| **Abilità comunicative** | | | | |
| 1. Come valuta la sua capacità di lettura in italiano? | 28 | III | 54%* | 4% |
| 2. Come valuta la sua capacità di scrittura in italiano? | 28 | III | 57%* | 11% |
| **Sostegno sociale** | | | | |
| 3. Con chi vive? | 29 | II | 86% | 0% |
| 4. Può contare su qualcuno quando ha bisogno di aiuto nelle attività quotidiane? | 29 | I | 72% | 0% |
| 5. Sente di avere bisogno di più aiuto nelle attività quotidiane? | 29 | II | 79% | 0% |
| 6. Può contare su qualcuno per gli spostamenti con i mezzi di trasporto (es. automobile, mezzi pubblici…)? | 29 | II | 93% | 0% |
| 7. Sente di avere bisogno di più aiuto per gli spostamenti con i mezzi di trasporto (es. automobile, mezzi pubblici…)? | 29 | I | 72% | 0% |
| 8. Può contare su qualcuno per la gestione della casa (es. pulizie, manutenzione…)? | 29 | II | 83% | 0% |
| 9. Sente di avere bisogno di più aiuto nella gestione della casa (es. pulizie, manutenzione…)? | 29 | II | 83% | 0% |
| 10. Sente il bisogno di parlare con più persone? | 29 | I | 83% | 0% |
| 11. Con quante persone trascorre il suo tempo abitualmente? | 29 | II | 90% | 0% |
| 12. Sente di avere bisogno di trascorrere più tempo con amici e familiari? | 29 | I | 83% | 0% |
| 13. Ha qualcuno con cui confidarsi o a cui chiedere consigli? | 29 | I | 76% | 0% |
| 14. Sente di avere bisogno di più sostegno in merito a questioni importanti (es. spese improvvise, decisioni mediche…)? | 29 | II | 72% | 0% |
| 15. È soddisfatto/a dei suoi rapporti familiari? | 29 | I | 90% | 0% |
| 16. Ha buoni rapporti con i suoi vicini di casa? | 29 | I | 79% | 0% |
| **Attività quotidiane in ambito sociale** | | | | |
| 17. Utilizza il telefono? | 29 | I | 72% | 0% |
| 18. Utilizza strumenti digitali (smartphone, tablet, pc) per comunicare (es. social media, videochiamate), informarsi (quotidiani online), ricevere supporto medico (telemedicina) e/o accedere ai servizi della pubblica amministrazione? | 29 | II^#^ | 86% | 0% |
| 19. Fa parte di organizzazioni di volontariato o altri gruppi? | 29 | I | 90% | 0% |
| 20. Sente di essere utile alla sua famiglia e/o alla comunità? | 29 | I | 76% | 0% |
| **Attività ricreative** | | | | |
| 21. Con quale frequenza incontra i suoi amici o familiari? | 29 | II | 90% | 0% |
| 22. Con quale frequenza si dedica ad attività da svolgere all’aria aperta (es. giardinaggio/orto, pesca, parco…)? | 29 | II | 83% | 0% |
| 23. Con quale frequenza si dedica ad attività da svolgere a casa (bricolage, cucina, parole crociate, lavoro a maglia, lettura…)? | 29 | II | 79% | 0% |
| 24. Con quale frequenza svolge attività fisica in compagnia (es. ballo, passeggiate, sport…)? | 29 | II | 86% | 0% |
| 25. Con quale frequenza visita circoli, luoghi religiosi, centri civici o altri centri di aggregazione sociale? | 29 | II | 93% | 0% |
| 26. Con quale frequenza prende parte ad attività culturali (es. università delle tre età, teatro, attività musicali, viaggi…)? | 29 | II^#^ | 79% | 0% |
| 27. Con quale frequenza gioca a carte o ad altri giochi di società? | 29 | II | 93% | 0% |
| 28. Si occupa di un animale da compagnia? | 29 | I | 79% | 0% |
| **Benessere psicologico** | | | | |
| 29. Sente di poter decidere liberamente sulle questioni importanti della sua vita? | 28 | III | 61%* | 0% |
| 30. Riscontra difficoltà nel gestire e mantenere relazioni con le persone significative della sua vita? | 29 | II | 76% | 0% |
| 31. Ritiene di avere relazioni profonde e basate sulla fiducia? | 29 | I | 86% | 0% |
| 32. Ha qualcuno che la faccia sentire amato/a e che si prenda cura di lei? | 29 | I | 76% | 0% |
| 33. Si sente soddisfatto/a della sua vita attualmente? | 29 | I | 76% | 0% |
| **Situazione socio-economica** | | | | |
| 34. Il suo reddito attualmente soddisfa le sue necessità? | 29 | I | 83% | 0% |
| 35. Ha una casa di proprietà? | 29 | I | 97% | 0% |
| 36. La sua abitazione è adeguata alle sue esigenze? | 29 | I | 90% | 0% |
| 37. Qual è il suo titolo di studio? | 29 | I | 90% | 0% |
| 38. Sente di avere adeguato accesso alle cure (studio medico di medicina generale, visite specialistiche, cure domiciliari e infermieristiche…)? | 29 | II^#^ | 86% | 0% |

***** A threshold of 50%+1 was considered acceptable for items requiring re-discussion

^#^ Item proposed by the panel during round I and approved during round II.
